# A Case Report of Eosinophilic Sialodochitis with Right Submandibular Sialolithiasis and Hyperattenuating Material Along Wharton’s Duct

**DOI:** 10.3390/reports9030272

**Published:** 2026-08-14

**Authors:** Tomohiro Kawasumi, Takao Hamamoto, Takashi Ishino, Tsutomu Ueda, Sachio Takeno

**Affiliations:** Department of Otorhinolaryngology, Head and Neck Surgery, Faculty of Medicine, Graduate School of Biomedical and Health Sciences, Hiroshima University, Hiroshima 734-8551, Japan; kwtm2022@hiroshima-u.ac.jp (T.K.); takao0320@hiroshima-u.ac.jp (T.H.); tishino@hiroshima-u.ac.jp (T.I.); uedatsu@hiroshima-u.ac.jp (T.U.)

**Keywords:** eosinophilic sialodochitis, Wharton’s duct, sialolithiasis, eosinophilic mucin, Charcot–Leyden crystals, high-attenuation mucus

## Abstract

**Background and Clinical Significance**: Eosinophilic sialodochitis (ES), also known as sialodochitis fibrinosa, is a rare disorder characterized by recurrent salivary gland swelling caused by intraductal eosinophilic mucous plugs. Typical histopathological findings include eosinophils and Charcot–Leyden crystals within ductal secretions, and characteristic imaging findings include salivary duct dilatation and glandular swelling. Although rare cases associated with sialolithiasis or calcification have been reported, high-attenuation material within the salivary duct on computed tomography (CT) has not been clearly described in ES. **Case Presentation**: A 56-year-old woman with allergic rhinitis presented with recurrent swelling and pain in the right submandibular area. CT and ultrasonography revealed a large sialolith in the right submandibular gland and dilatation of Wharton’s duct. She underwent right submandibular gland excision for presumed chronic obstructive submandibular sialadenitis with a sialolith. Soon after surgery, she developed recurrent swelling of the right floor of the mouth, and CT showed persistent high-attenuation material along Wharton’s duct without residual sialolith. Ductal massage discharged a brownish gelatinous material. Histopathological examination revealed numerous eosinophils and Charcot–Leyden crystals in both the discharged mucous plug and decalcified sialolith, fulfilling Baer’s diagnostic criteria for ES. Physical extraction and anti-allergic medications were insufficient, whereas ductal irrigation with saline and triamcinolone acetonide markedly reduced mucous plug discharge. Symptoms were controlled during 18 months of follow-up. **Conclusions**: Retained eosinophilic mucin in ES may appear as high-attenuation ductal material on CT and contribute to salivary stasis and sialolith formation.

## 1. Introduction and Clinical Significance

Eosinophilic sialodochitis (ES), also known as sialodochitis fibrinosa, is a rare disease characterized by the formation of fibrinous plugs within the salivary ducts, resulting in impaired salivary flow and recurrent swelling of the salivary glands. Histopathological examination typically reveals mucous plugs containing eosinophils and Charcot–Leyden crystals, as well as eosinophilic infiltration around the major salivary gland ducts [1]. A recent review reported that most patients with ES were atopic and that 75.4% had allergic rhinitis [2]. Elevated serum IgE levels and peripheral blood eosinophilia in some patients suggest possible involvement of type I allergic mechanisms. Baer’s criteria (Table 1), which include clinical and pathological findings, are commonly used for the diagnosis. Characteristic imaging findings of ES include salivary duct dilatation and swelling of the salivary glands [3,4,5]. Rare cases associated with sialoliths or calcifications have also been reported [6]. However, to our knowledge, no previous report has clearly described a high-attenuation area along the salivary duct on computed tomography (CT) suggestive of eosinophilic mucin retention within the duct in ES.

Here, we report a case of ES that initially presented as right submandibular sialolithiasis and showed a high-attenuation area within the right Wharton’s duct on preoperative CT. In this case, histology revealed numerous eosinophils and Charcot–Leyden crystals not only within the lumen of Wharton’s duct but also within the sialolith. This case may provide useful insights into the relationship between eosinophilic inflammation, CT findings, and sialolith formation in patients with ES.

## 2. Case Presentation

The patient was a 56-year-old woman who visited a local clinic with a chief complaint of recurrent swelling and pain in the right submandibular region. Her medical history included allergic rhinitis. Physical examination revealed right submandibular swelling with tenderness on palpation. Laboratory tests showed a white blood cell count of 8460 cells/µL, an absolute eosinophil count of 670 cells/µL (7.9%), and a serum total IgE level of 114 IU/mL. Serum antigen-specific IgE testing using the View Allergy 39 (a multiple-allergen-specific IgE screening test) showed positivity for Japanese cedar pollen and house dust mites, with values of 1.49 IU/mL and 10.61 IU/mL, respectively. The complete allergen-specific IgE results are presented in Supplementary Table S1. Laboratory findings ruled out differential diagnoses associated with recurrent submandibular swelling, including Sjögren’s syndrome, IgG4-related sialadenitis, and Kimura’s disease (Table 2).

**Figure 1:**
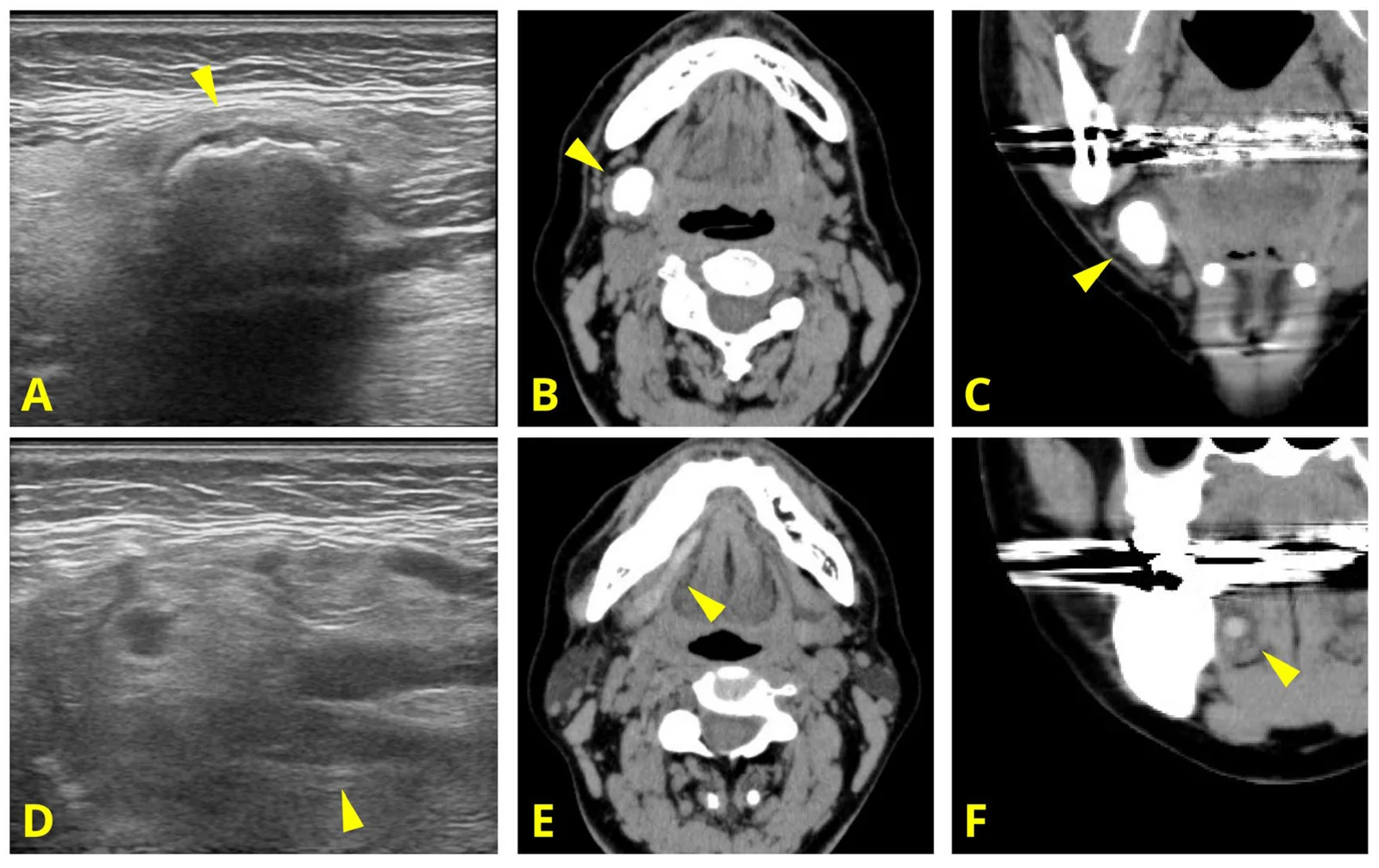
Computed tomography (CT) and ultrasonography findings before right submandibular gland excision: (**A**,**B**) A calcified lesion measuring 11 × 14 × 16 mm was observed in the right submandibular gland (arrowheads). (**C**) A calcified lesion with acoustic shadowing was observed in the right submandibular gland (arrowhead). (**D**,**E**) The right Wharton’s duct was dilated, and a high-attenuation area, with a mean CT value of 110 HU, was observed along the duct lumen. Edematous changes were present in the tissue surrounding Wharton’s duct (arrowhead). (**F**) Dilatation of the right Wharton’s duct was observed. No apparent calcification was detected within the duct (arrowhead).

Cervical ultrasonography (US) revealed a calcified lesion with acoustic shadowing in the right submandibular gland (Figure 1A). CT confirmed an 11 mm × 14 mm × 16 mm sialolith in the gland (Figure 1B,C). Both US and CT revealed dilatation of Wharton’s duct (Figure 1D–F). Based on these findings, intraductal pus retention secondary to chronic obstructive submandibular sialadenitis associated with sialolithiasis was initially suspected. Because spontaneous expulsion or isolated removal was considered difficult owing to the location and size of the stone, a right submandibular gland resection was performed.

**Figure 2:**
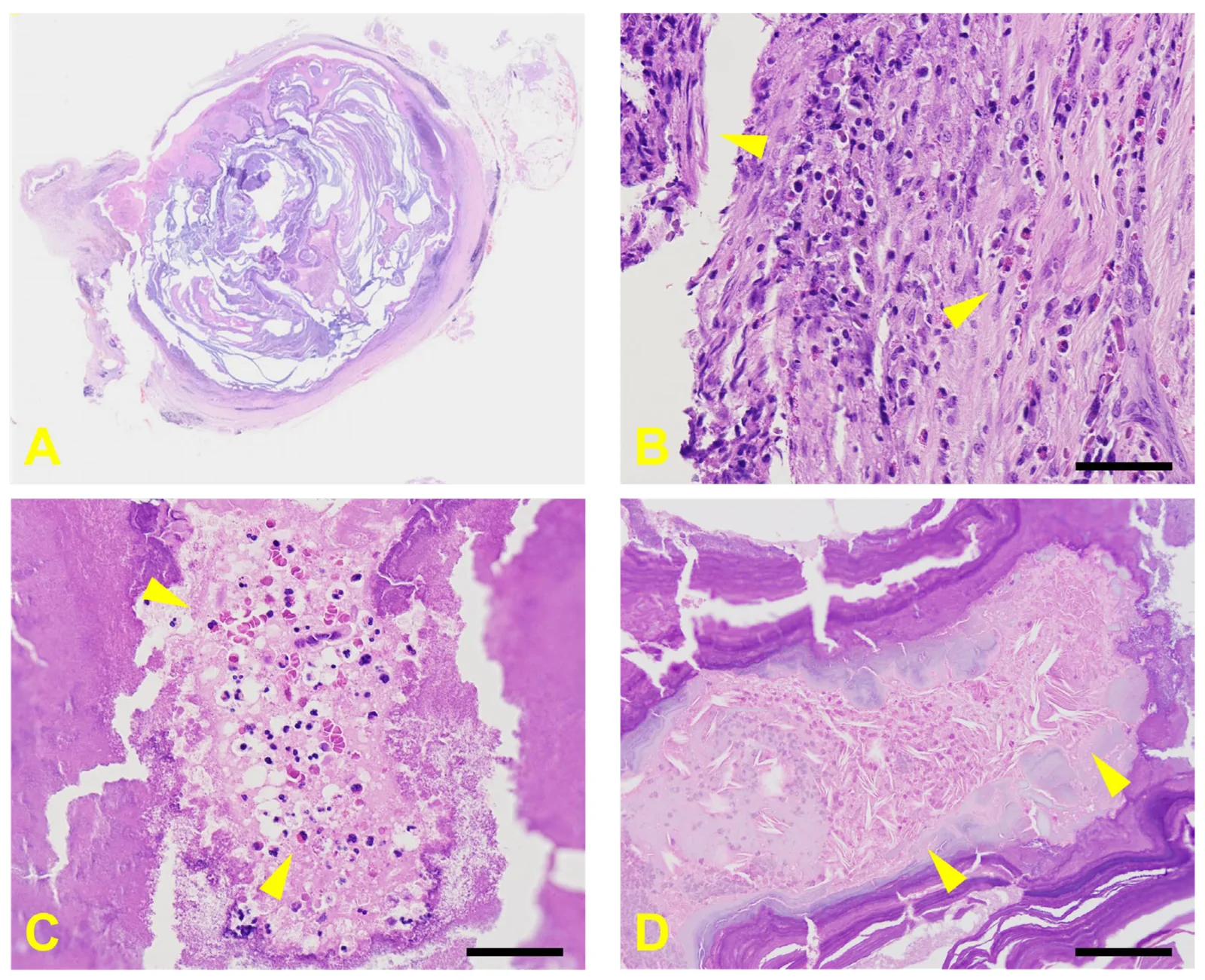
Pathological findings of the sialolith: (**A**) Gross appearance of the sialolith located at the hilum of the submandibular gland. (**B**) Hematoxylin and eosin (H&E) staining showed inflammatory cell infiltration, mainly composed of lymphocytes, with fibrotic changes, desquamated fragments of ductal epithelium, and focal eosinophilic infiltration in the ductal wall (arrowheads; scale bar = 50 μm). (**C**,**D**) Numerous eosinophils and Charcot–Leyden crystals were observed predominantly around the sialolith, with some also present within the sialolith (arrowheads; (**C**) scale bar = 50 μm; (**D**) scale bar = 100 μm).

Histopathological examination of the resected specimen using hematoxylin and eosin staining revealed a sialolith at the hilum of Wharton’s duct (Figure 2A). Periductal fibrosis, fragments of ductal epithelium, and lymphocytic and eosinophilic infiltration around the ducts were also observed (Figure 2B). Notably, after decalcification of the sialolith, numerous eosinophils and Charcot–Leyden crystals were observed both around and within the sialolith (Figure 2C,D).

**Figure 3:**
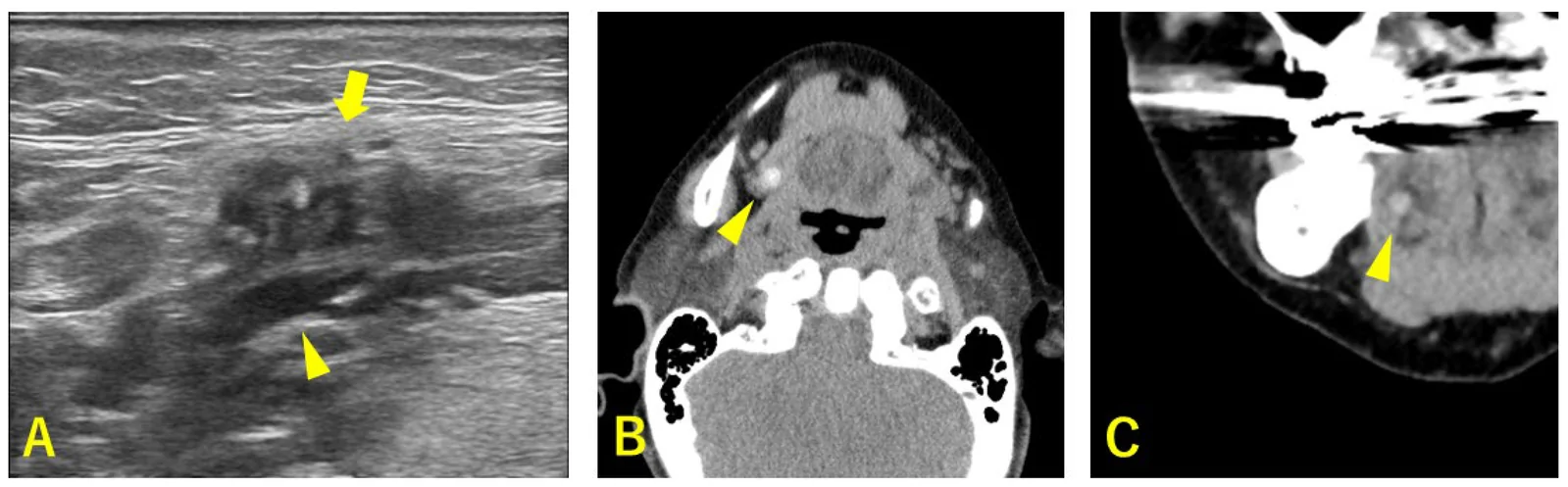
Computed tomography (CT) and ultrasonography findings after right submandibular gland excision: (**A**) Ultrasonography showed no residual calculi (arrow) and persistent dilatation of Wharton’s duct (arrowhead). (**B**,**C**) The high-attenuation area within the right Wharton’s duct, with a mean CT value of 159 HU, and the surrounding edematous changes were slightly reduced but remained present (arrowheads).

Soon after surgery, the patient developed frequent pain and swelling of the right floor of the mouth, with expulsion of a brownish jelly-like secretion from Wharton’s duct. US and CT revealed dilatation of the right Wharton’s duct, and CT showed a high-attenuation area within the duct, with no obvious residual sialolith (Figure 3A–C). Similar findings were retrospectively identified on preoperative CT, and ductal dilatation was considered unlikely to be caused by the sialolith itself or by chronic obstructive submandibular sialadenitis.

**Figure 4:**
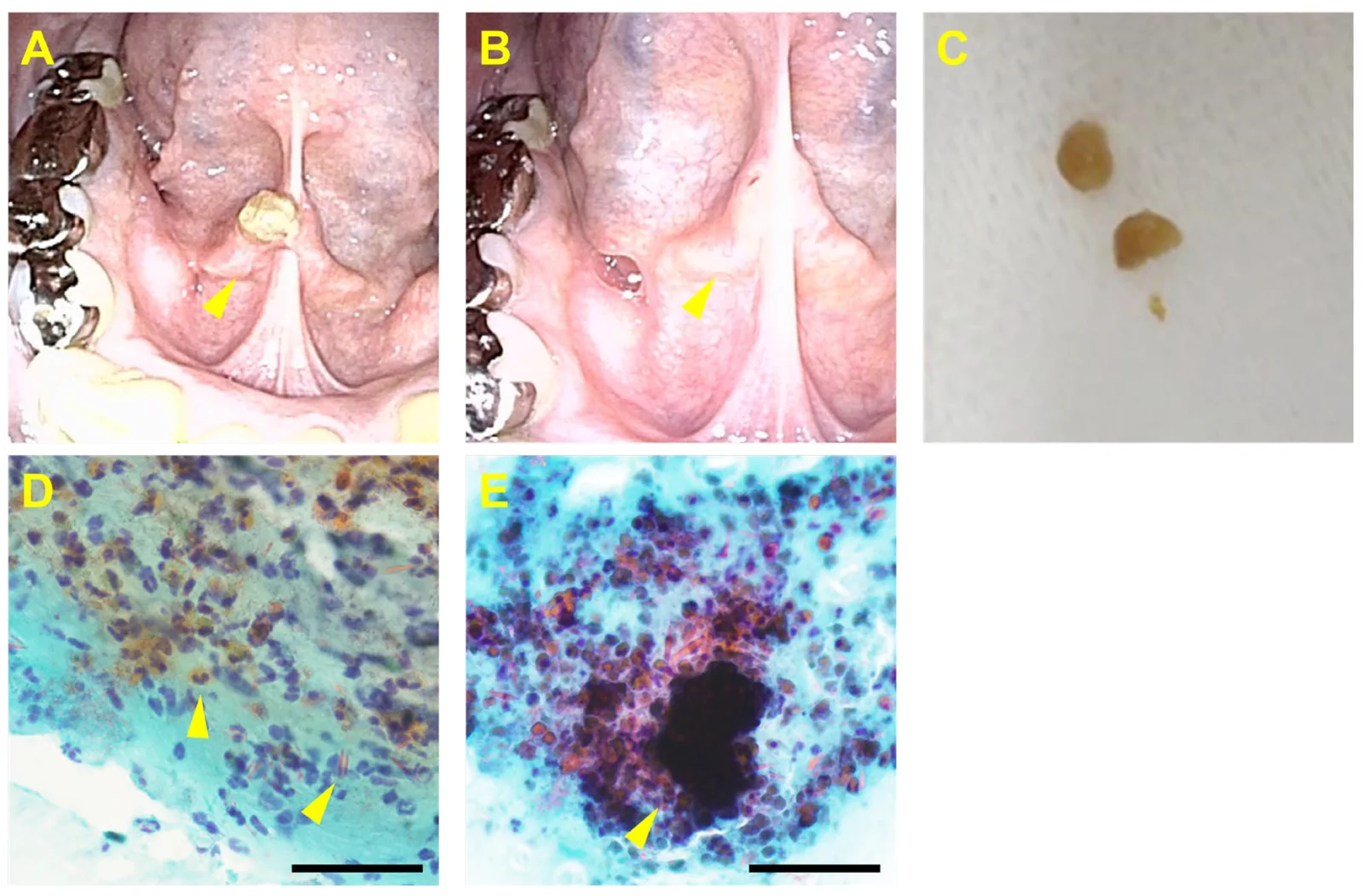
Mucous plug obstruction of the salivary duct: (**A**) A mucous plug was discharged from the right Wharton’s duct (arrowhead). (**B**) After irrigation of Wharton’s duct, dilation of the ductal orifice was noted (arrowhead). (**C**) The mucous plug was yellowish-brown and gelatinous, with no apparent residual sialoliths. (**D**) Numerous eosinophils and Charcot–Leyden crystals were observed in salivary mucins (arrowheads; scale bar = 50 μm). (**E**) Anuclear crystalline structures suggestive of sialomicroliths were observed at sites of eosinophil aggregation (arrowhead; scale bar = 100 μm).

Intraoral examination revealed a mucous plug extruding from the right Wharton’s duct (Figure 4A). The plug was collected as a specimen, and ductal cannulation with saline irrigation revealed dilatation of the ductal orifice (Figure 4B,C). Papanicolaou staining of the mucous plug revealed numerous eosinophils and Charcot–Leyden crystals (Figure 4D). Clusters of eosinophils and an anucleate crystal-like structure were also observed in the mucous plug (Figure 4E). As the clinical and histological findings met Baer’s criteria, with the exception of the IgE level (Table 1), the patient was diagnosed with eosinophilic sialodochitis.

Physical extraction of mucous plugs was performed, and anti-allergic medications, including bilastine (20 mg) and montelukast (10 mg), were administered for 3 months; however, their efficacy was limited. Therefore, ductal irrigation with saline and corticosteroids was initiated. The irrigation consisted of 2 mL of normal saline and 0.1 mL of triamcinolone acetonide 40 mg/mL. After steroid irrigation, the mucous plug discharge markedly decreased. Following two additional steroid irrigations at 6-month intervals, the patient’s symptoms remained well controlled with self-extraction of residual mucous plugs and saline irrigation every 3 months during 18 months of follow-up. The clinical course is summarized in Supplementary Figure S1.

## 3. Discussion

Eosinophilic sialodochitis (ES) is a rare inflammatory disease of the salivary glands that primarily affects the excretory ducts of the major salivary glands. ES was first described by Kussmaul in 1879. It is characterized by recurrent painful swelling of the salivary glands and mucous plugs containing leukocytes and Charcot–Leyden crystals. Baer’s criteria (Table 1) are the most commonly used diagnostic criteria [1]. Recent reviews, including a systematic review, have summarized the clinical features, diagnostic criteria, and treatment outcomes of ES [2,3].

ES is reported to occur more often in women and commonly involves the bilateral parotid glands, although unilateral cases and cases involving the submandibular gland have also been described [2,3]. In the present case, the patient had recurrent swelling of the right floor of the mouth and discharge of viscous secretions from the orifice of Wharton’s duct, containing numerous eosinophils and Charcot–Leyden crystals. The differential diagnoses included IgG4-related sialadenitis, Sjögren’s syndrome, Kimura’s disease, and chronic obstructive submandibular sialadenitis. IgG4-related sialadenitis and Sjögren’s syndrome were considered unlikely because the serum IgG4 level was within the normal range, and anti-SS-A/Ro and anti-SS-B/La antibodies were negative. Kimura’s disease was also considered unlikely because the serum total IgE level was not markedly elevated and no characteristic subcutaneous mass or lymphadenopathy was observed. The patient was initially diagnosed with chronic obstructive submandibular sialadenitis associated with a sialolith and underwent submandibular gland excision. However, based on the postoperative clinical course and histopathological findings of eosinophil-rich mucous plugs discharged from Wharton’s duct, the diagnosis was retrospectively revised to eosinophilic sialodochitis. The patient met the mandatory diagnostic features of Baer’s criteria [1], although the serum total IgE level was not elevated.

In the present case, mucous plug-related symptoms became clinically apparent after submandibular gland resection. Previous reports have described submandibular gland excision as a surgical treatment for ES involving the submandibular gland; however, mucous plug symptoms have also become evident postoperatively [6,7]. In contrast, long-term disease control has been achieved in cases treated with additional or simultaneous resection of the affected salivary duct [7,8]. These findings suggest that the salivary duct, rather than the gland itself, may be the primary site involved in the pathogenesis of ES. A recent review summarized treatment options for ES, including conservative ductal interventions (saline and/or steroid irrigation, mechanical dilatation, and drain placement), medical therapy (antihistamines, leukotriene receptor antagonists, and systemic corticosteroids), and surgical excision of the involved gland and duct [2,3]. Among these approaches, conservative ductal interventions have been associated with the highest long-term success rates among currently available treatments. Recently developed disease-specific instruments, such as the Chronic Sialadenitis Outcome Test-14, may enable a more standardized evaluation of symptoms, functional limitations, quality of life, and treatment response [9]. Although such instruments were not used in the present case, they should be considered in future studies. The favorable outcomes of local therapies targeting the salivary duct further support the central role of ductal involvement in the pathogenesis of ES. In the present case, submandibular gland resection was considered unavoidable because of the presence of a large intraglandular sialolith. At the time of surgery, ES had not yet been diagnosed; therefore, Wharton’s duct was preserved. After the postoperative diagnosis of ES, ductal resection was considered; however, the patient declined further surgery. Conservative treatment was therefore continued, and ductal resection will be considered if the disease becomes refractory.

An important feature of the present case was the high-attenuation area along the right Wharton’s duct on the CT scan. Viscous eosinophilic mucin in type 2 inflammatory diseases, such as allergic bronchopulmonary aspergillosis (ABPA) and eosinophilic chronic rhinosinusitis (ECRS), appears as high-attenuation areas on CT [10,11]. In particular, as a characteristic finding of ABPA, high-attenuation mucus (HAM) is defined as mucus with a CT value of 70 Hounsfield units (HUs) or higher, which helps distinguish it from mucus plugs associated with other diseases [10]. In contrast, the imaging findings of ES reported to date mainly include ductal dilatation and salivary gland swelling [4,5]. In the present case, the high-attenuation area along Wharton’s duct corresponded to the site from which viscous secretions containing eosinophils and Charcot–Leyden crystals were discharged. The mucous plug was discharged immediately after postoperative CT and contained numerous eosinophils and Charcot–Leyden crystals, supporting a close temporal and anatomical correlation with hyperattenuating intraductal material. The attenuation value of the intraductal material was consistently 70 HU or higher both before and after surgery. Therefore, this CT finding may be considered analogous to the high-attenuation mucus reported in eosinophilic airway diseases.

In many cases of ES, concomitant allergic disease, elevated eosinophil counts and IgE levels, and numerous eosinophils in ductal secretions have been reported [12]. Eosinophilic inflammation associated with type I allergy is considered to contribute to the pathogenesis of this disease. In the present case, although the total IgE level was not markedly elevated, the patient had peripheral blood eosinophilia and a history of allergic rhinitis. Recent studies have suggested that eosinophilic ETosis (EETosis) may be involved in the pathogenesis of ES [7]. ETosis is a form of cell death observed in granulocytes, including neutrophils and eosinophils, in which extracellularly released chromatin and granule proteins form web-like structures [13,14]. Previous studies have suggested that EETosis may contribute to the formation of viscous eosinophilic mucin in ES and other type 2 inflammatory diseases [7,14,15,16]. In the present case, the presence of numerous eosinophils and Charcot–Leyden crystals in the viscous mucus plug was compatible with eosinophilic inflammation. However, EETosis has not been directly demonstrated, and its involvement remains hypothetical.

Another characteristic feature of this case is that ES was identified during the evaluation of right submandibular sialolithiasis in the patient. ES associated with sialolithiasis is rare, with only a limited number of cases reported in the literature [6]. Although the mechanism of sialolith formation remains unclear, the aggregation of sialomicroliths [17,18], anatomical factors of the salivary ducts [19,20], and changes in the biochemical composition of saliva [21] are considered to be involved in its formation. Sialomicrolith itself has been reported in normal salivary glands of asymptomatic individuals [20]. A recent ultrastructural study showed that sialoliths consist of three layers: a central nidus, an intermediate compact zone, and a peripheral multilayered zone [22]. The authors proposed that bacterial infection and biofilm formation in the central nidus, promoted by ductal congestion, may facilitate the aggregation of sialomicroliths and subsequent sialolith formation. In addition, neutrophil ETosis has been suggested to promote calcium-based crystal formation [23]. In the present case, sialomicrolith-like structures were observed within mucous plugs, along with eosinophil clusters and Charcot–Leyden crystals. The coexistence of eosinophil-rich mucous plugs, Charcot–Leyden crystals, sialomicrolith-like structures, and a sialolith raises the possibility of an association between eosinophilic inflammation and sialolith formation. However, the present case did not establish a causal relationship or the direction of this association.

This study has several limitations. First, this report describes only a single case; therefore, a causal relationship between eosinophilic inflammation and sialolith formation cannot be established. Second, although the sialolith was examined histopathologically after decalcification, no structural, mineralogical, biochemical, or ultrastructural characterization was performed. Furthermore, direct pathological continuity between the sialolith removed from the submandibular gland and the secretions discharged from Wharton’s duct postoperatively could not be demonstrated. Therefore, the relationship between eosinophilic inflammation and sialolith formation remains speculative, and chronic obstruction caused by the sialolith may have contributed to the observed inflammatory changes. Third, the involvement of eosinophilic ETosis was not confirmed by immunohistochemistry and was inferred only from the pathological findings. These issues should be addressed in future research. Fourth, no direct biochemical analysis of the mucous plug composition was performed; therefore, its eosinophilic nature was inferred from cytological and histopathological findings. Fifth, treatment response was assessed descriptively, and no validated patient-reported symptom or quality of life instrument was administered. Future studies should incorporate disease-specific patient-reported outcome measures to permit the standardized assessment of therapeutic efficacy.

## 4. Conclusions

We report a case of ES identified during the evaluation of right submandibular sialolithiasis, which showed a high-attenuation area along the right Wharton’s duct on CT. The presence of numerous eosinophils and Charcot–Leyden crystals in the discharged material suggests that eosinophilic mucin retained within the salivary duct may appear as a high-attenuation area on CT. These findings may have contributed to salivary stasis and sialolith formation. This case provides useful insights into the imaging findings and pathophysiology of ES.

## Figures and Tables

**Table 1:** Baer’s diagnostic criteria for eosinophilic sialodochitis.

Criteria
(1) Recurrent paroxysmal swelling of the major salivary glands;
(2) Salivary duct mucus plugs containing numerous eosinophils;
(3) Peripheral blood eosinophilia and elevated IgE level;
(4) Associated atopic disease;
(5) Ductal dilatation and occasional focal narrowing of the major salivary gland ducts;
(6) Periductal eosinophil- and lymphocyte-rich inflammation and fibrosis with associated reactive ductal epithelial cells;
(7) Failure to satisfy the diagnostic criteria of IgG4-related disease.

NOTE: Mandatory features of eosinophilic sialodochitis include satisfying criteria 1 and 2 or criteria 1, 6, and 7. IgE, immunoglobulin E; IgG4, immunoglobulin G4.

**Table 2:** Laboratory data in the present case.

Parameter	Value	Parameter	Value
White blood cells	8460/µL	Total protein	7.3 g/dL
Neutrophils	3900/µL (46.1%)	Albumin	4.3 g/dL
Lymphocytes	3180/µL (37.6%)	Total bilirubin	0.3 mg/dL
Monocytes	650/µL (7.7%)	Aspartate aminotransferase	20 U/L
Eosinophils	670/µL (7.9%)	Alanine aminotransferase	34 U/L
Basophils	60/µL (0.7%)	Lactate dehydrogenase	148 U/L
Red blood cells	448 × 10^4^/µL	Amylase	63 U/L
Hemoglobin	13.6 g/dL	Sodium	141 mmol/L
Hematocrit	42.2%	Potassium	4.0 mmol/L
Platelets	30.8 × 10^4^/µL	Chloride	106 mmol/L
		Calcium	9.5 mg/dL
		Blood urea nitrogen	15.4 mg/dL
		Creatinine	0.62 mg/dL
		IgG	1148 mg/dL
		IgE	114 IU/mL
		IgG4	20.9 mg/dL
		Anti-SS-A/Ro antibody	<1.0 U/mL
		Anti-SS-B/La antibody	<1.0 U/mL

Ig, immunoglobulin; SS, Sjögren syndrome.

## Data Availability

The data presented in this article are available from the corresponding author upon reasonable request. Public sharing of patient data is restricted to protect patient privacy.

## Supplementary Materials

The following supporting information can be downloaded at https://www.mdpi.com/article/10.3390/reports9030272/s1, Table S1. Results of serum allergen-specific IgE testing using the View Allergy 39 multiple-allergen screening test. Figure S1. Clinical timeline of the present case.

## Abbreviations

The following abbreviations are used in this manuscript:

ABPA	Allergic bronchopulmonary aspergillosis
ECRS	Eosinophilic chronic rhinosinusitis
EETosis	Eosinophilic extracellular trap cell death
ES	Eosinophilic sialodochitis
HAM	High-attenuation mucus
H&E	Hematoxylin and eosin
HUs	Hounsfield units
US	Ultrasonography
