# Supplementary material for: A Case Report of Eosinophilic Sialodochitis with Right Submandibular Sialolithiasis and Hyperattenuating Material Along Wharton’s Duct
Source: Reports (MDPI). 2026 Aug 14;9(3):272. doi: 10.3390/reports9030272 (PMC13510531; doi:10.3390/reports9030272)
Supplement: Supplementary file 1 [file reports-09-00272-s001.zip › reports-4457871-supplementary.pdf]

Supplemental Data

Supplementary Table S1. Results of serum allergen-specific IgE testing using the View Allergy 39 multiple-allergen screening test.

Allergen	Specific IgE (IU/mL)	Interpretation
Orchard grass	<0.27	Negative
Timothy grass	<0.27	Negative
Ragweed	<0.27	Negative
Mugwort	<0.27	Negative
Japanese cedar	1.49	Positive
Japanese cypress	0.35	Positive
Alder	<0.27	Negative
Birch	<0.27	Negative
House dust mite 1	10.61	Positive
House dust 1	10.56	Positive
Candida	<0.27	Negative
Alternaria	0.31	Positive
Aspergillus	<0.27	Negative
Malassezia	<0.27	Negative
Cat dander	<0.27	Negative
Dog dander	<0.27	Negative
Wheat	<0.27	Negative
Soybean	<0.27	Negative
Rice	<0.27	Negative
Sesame	<0.27	Negative
Tuna	<0.27	Negative
Salmon	<0.27	Negative
Mackerel	<0.27	Negative
Shrimp	<0.27	Negative
Crab	<0.27	Negative
Milk	<0.27	Negative
Beef	<0.27	Negative
Chicken	<0.27	Negative
Pork	<0.27	Negative
Egg white	<0.27	Negative
Ovomucoid	<0.27	Negative
Buckwheat	<0.27	Negative
Peanut	<0.27	Negative
Apple	<0.27	Negative
Kiwi fruit	<0.27	Negative
Banana	<0.27	Negative
Latex	<0.27	Negative
Cockroach	<0.27	Negative
Moth	0.29	Positive

Note: Values reported as below the assay threshold are shown as <0.27 IU/mL. Results flagged as high by the testing laboratory are categorized as positive. IgE, immunoglobulin E.

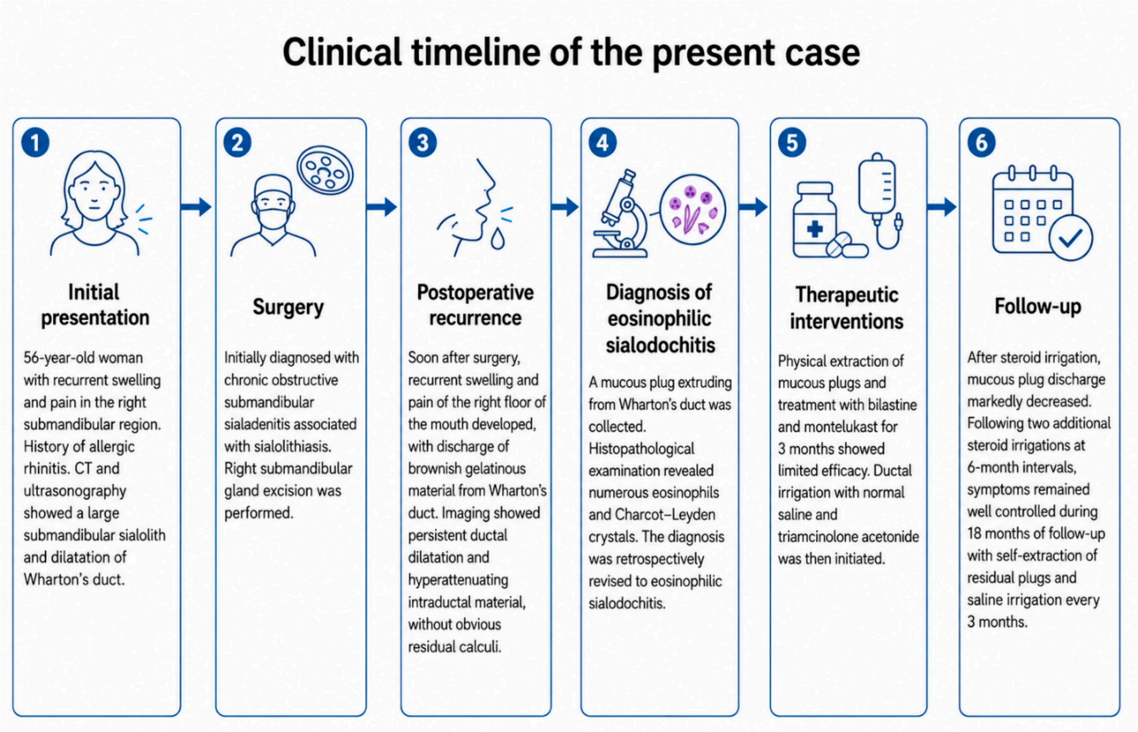

Supplementary Figure S1. Clinical timeline of the present case. The timeline summarizes the initial presentation, initial diagnosis and surgery, postoperative recurrence, diagnosis of eosinophilic sialodochitis, therapeutic interventions, and 18-month follow-up.
